# A256 NAVIGATING IBD CARE : HEALTHCARE PROVIDERS’ INSIGHTS ON IMPLEMENTATION OF NURSE NAVIGATOR-LED IBD FLARE PATHWAY

**DOI:** 10.1093/jcag/gwad061.256

**Published:** 2024-02-14

**Authors:** J Reuangrith, J Jones, N Rohatinsky, G Nguyen

**Affiliations:** Dalhousie University, Halifax, NS, Canada; Dalhousie University, Halifax, NS, Canada; University of Saskatchewan, Saskatoon, SK, Canada; University of Toronto, Toronto, ON, Canada

## Abstract

**Background:**

Disparities in access to healthcare for persons living with inflammatory bowel disease (IBD) persist. An IBD nurse navigational role promises to improve access to evidence-based specialist care by minimizing inequities in access to care. Little is known about how to implement a provincial nurse navigational role in the Canadian health system.

**Aims:**

This study aims to explore healthcare providers' perceptions of barriers and facilitators to the implementation of an evidence-based IBD flare pathway through a provincial nurse navigational role.

**Methods:**

This was a national qualitative study performed in collaboration with the CCC PACE Program, in which two virtual semi-structured group interviews were conducted in 2022. One group interview was with six IBD nurses from three provinces, and the second was with five gastroenterologists from five provinces. Participants were purposefully recruited. The Consolidated Framework for Implementation Research (CFIR) and the Capability, Opportunity, Motivation, and Behaviour (COM-B) frameworks informed the interview script development. Data were transcribed verbatim and subjected to content analysis, with major themes categorized into the COM-B domains. The results of preliminary data analysis are presented.

**Results:**

While IBD nurses emphasized the critical role of effective communication tools, standardized referral systems, strong administrative support and patient education and engagement as facilitators, they also highlighted challenges like patient resistance, variability of training and the necessity for tailored patient care. Other barriers identified were the medical complexity of managing overlapping conditions, remote vs in-person considerations and the perception and definition of a flare. On the other hand, IBD-focused gastroenterologists emphasized the potential of integrating pathways into electronic medical records and the value of empowering nurse navigators. However, they pointed out concerns regarding the inherent variability in disease presentation, entrenched medical beliefs, comfort levels with nurse-led pathways and the inherent diversity in medical practices. Both groups identified resource constraints as a barrier and an effective triage process as a facilitator.

**Conclusions:**

This study sheds light on the multifaceted perceptions of healthcare providers concerning the implementation of an evidence-based IBD flare pathway through a provincial nurse navigational role. These insights underline the complexity of implementing a nurse navigational role and the need for targeted interventions and resources to address the identified barriers while leveraging the facilitators.

**Funding Agencies:**

CCC

